# Solid-state fermentation as a strategy for improvement of bioactive properties of the plant-based food resources

**DOI:** 10.1186/s40643-025-00981-7

**Published:** 2025-12-03

**Authors:** Sirma Yegin

**Affiliations:** Department of Food Engineering, Ege University, Bornova, 35100 Izmir, Turkey

**Keywords:** Solid-state fermentation, Bioactive phenolics, Nitrogenous bioactive compounds, Enzymes, Biorefinery

## Abstract

Currently, there is growing interest in gaining healthy eating habits through the consumption of sufficient amounts of natural bioactive compounds such as phenolic compounds and peptides. The major reason behind this interest is that the incorporation of such bioactive compounds into the diet exhibits great potential in the reduction of various types of chronic disease risks. Therefore, several strategies have been developed for the production and isolation of such compounds. Plant-based materials are the main source of natural bioactive compounds. However, the low concentration and the inactive form of these compounds in natural plant-based dietary resources appear as a limiting factor in most cases. Solid-state fermentation is a promising process for the generation and recovery of various high-value bioproducts. It has received more attention due to its exceptional potential to overcome all those limitations. This review provides an overview of various aspects of solid-state fermentation, including historical background, key microbial features, critical process variables, and cultivation systems. Furthermore, the potential of solid-state fermentation on the production of both phenolic compounds and nitrogenous bioactive compounds is described in detail by gathering the previous experiences and knowledge with the additional focus on the biorefinery concept.

**Graphical abstract:**

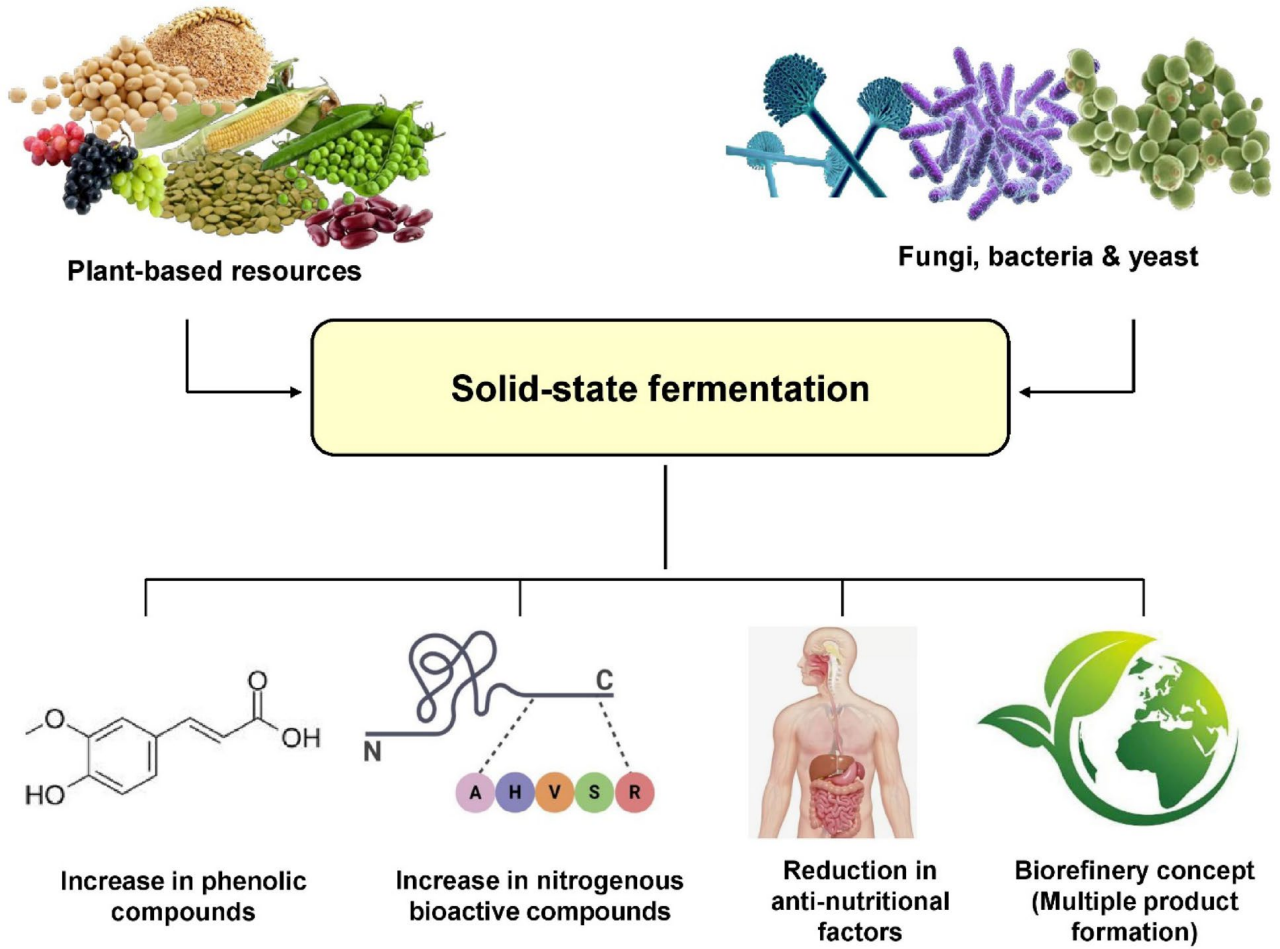

## Introduction

Nowadays, public awareness of health maintenance through the consumption of an adequate amount of natural bioactive compounds (e.g., phenolic compounds and peptides) has increased to a great extent. Consequently, several studies have been directed to this field with the focus on isolation and elucidation of health-promoting effects of these compounds. The main natural sources of bioactive compounds are plant-based materials, although they can be obtained from diverse resources, including animal-derived materials, fungi, and marine organisms. The pivotal biological activities that these compounds possess can range from antioxidant and antimicrobial to anti-inflammatory and anticancer activities. In general, these compounds are already being taken into the human body through consumption of plant-based foods in the diet. However, their low concentration in natural dietary resources remains a limiting factor in reaching the desired level of health-promoting effect in most cases. Therefore, it is necessary to develop new strategies to increase their concentrations and bioavailabilities.

**Fig. 1:**
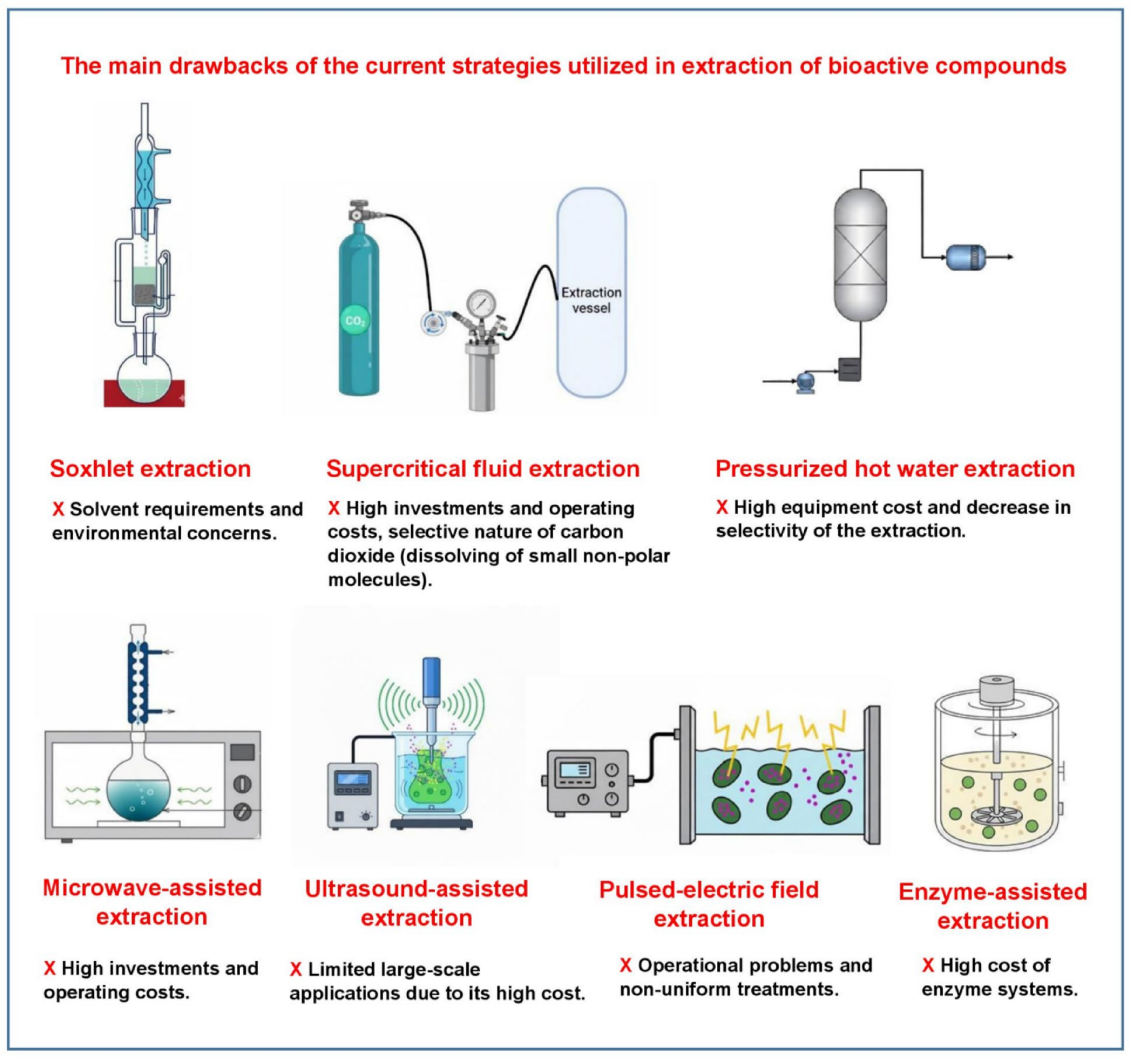
The main drawbacks of the current techniques utilized for extraction of bioactive compounds

Plant-based natural compounds possessing bioactive potential are usually synthesized in small quantities, which have commonly been entrapped within very complex matrices in plant tissues. The widely utilized techniques for extraction of these compounds are: (i) Soxhlet extraction, (ii) supercritical fluid extraction, (iii) pressurized hot water extraction, (iv) microwave-assisted extraction, (v) ultrasound-assisted extraction, (vi) pulsed-electric field extraction, and (vii) enzyme-assisted extraction. These techniques are high cost, labor-intensive, and time-consuming. The main drawback of each of these techniques is summarized in Fig. 1. Moreover, some of these techniques may result in degradation of the bioactive compounds, mainly phenolic compounds (Azmir et al. 2013; Kumar et al. 2021). The waste generated at the end of these processes also leads to environmental concerns. Although the enzyme-assisted extraction technique is an environmentally friendly option leading to less degradation with better bioavailability among the above-mentioned techniques (Kumar et al. 2021), utilization of the commercial multi-enzyme systems for this purpose is not economically feasible. Fermentative processes exhibit great potential as an alternative strategy enabling in situ production of various enzymes, which can provide the same or even better function than the commercial enzyme preparations. Solid-state fermentation has widely been used for the simultaneous production of multiple microbial enzymes from plant-based materials, especially agricultural wastes. The enzymes produced during a solid-state fermentation process are the key microbial products for the generation of bioactive compounds. Some of the hydrolytic enzymes (e.g., cellulase and xylanase) produced during a solid-state fermentation process can degrade the structural components of the plant tissue and facilitate the release of the entrapped bioactive compound(s) within the lignocellulosic structure. Oxidative enzymes, such as laccase enzymes, function in the biotransformation of lignin into bioactive compound(s). Protease enzymes take a role in the production of bioactive peptides by the hydrolysis of plant proteins during fermentation. In addition to the role of enzymes in generating bioactive compounds, the microbial strains can directly produce some bioactive compounds as a result of their own metabolism, such as carotenoids (β-carotene) (Roadjanakamolson and Suntornsuk 2010) and polysaccharides (e.g., levan) (Saeed et al. 2024) during solid-state fermentation of agricultural materials.

Currently, several informative reviews exist in literature regarding the potential of solid-state fermentation in the improvement of the bioactive properties of agricultural materials, including some cereal varieties. Most of these studies provided information on a specific class of compounds: the bioactive phenolics (Dey et al. 2016; Roasa et al. 2021; Martins et al. 2011). However, solid-state fermentation also has influence on protein digestibility, thereby leading to the production of peptides and amino acids with various bioactivities (Wang et al. 2023). Solid-state fermentation can also be utilized within the biorefinery concept for the production and sequential recovery of multiple products with bioactivities. The objective of the present review is to provide a wider perspective on solid-state fermentation of plant-based resources for the production/release of bioactive compounds by compiling prior experiences on both phenolic compounds and nitrogenous bioactives, with an additional emphasis on the biorefinery concept.

## Solid-state fermentation

### Definition and historical aspects

Solid-state fermentation is a bioprocess in which microorganisms grow on moist solid material(s) without the presence of free-flowing liquid. Although many researchers considered “solid-state fermentation” and “solid substrate fermentation” as the same terms, in fact they are not. In solid substrate fermentation, the inert solid material functions both as a support and as a nutrient source, mainly a carbon source, while in solid-state fermentation, the solid material does not necessarily need to be a nutrient source; it can be a natural substrate or an inert support. To date, the term “solid-state fermentation” is being used for any kind of solid substrate, either natural or an impregnated inert support. Impregnated synthetic inert supports usually provide more controllable conditions, enabling better kinetic studies due to their more homogenous structure as compared to the natural complex solid materials (e.g., agricultural wastes) (Krishna 2005).

According to the archeological discoveries, solid-state fermentation was first used long ago, dating back to approximately 2600 BC in bread making by Egyptians (Manan and Webb 2017a). It has further been employed in the manufacturing of traditional fermented food products, such as mold-ripened cheese. Koji is another product prepared by fermentation of mainly steamed rice by *Aspergillus oryzae*. The koji preparation process is also one of the oldest attempts of solid-state fermentation that is specific to China and Japan (Krishna 2005). There are various other region-specific traditional food products manufactured by utilizing solid-state fermentation. The solid substrates and the microbial strains involved in the manufacturing of these regional products are summarized in Table 1. The enzymes produced by the microbial strains utilized in these product formulations are the main microbial metabolites taking significant roles in the development of the desired sensory properties for these foods.

**Table 1:** Traditional food products manufactured by solid state fermentation

Name	Substrate	Main microbial strains involved	Originated region	Reference
Koji (grain form)	Rice	*Aspergillus oryzae*	China	Krishna 2005
Tempeh (cake form)	Soybean	*Rhizopus* sp. (*R*. *oligosporus*, *R*. *oryzae* & *R*. *stolonifera*)	Indonesia & Malaysia	Teoh et al. 2024
Miso (paste form)	Soybean	*A*. *oryzae*	Japan	Wang et al. 2019
Baijiu (distilled spirt)	Sorghum or a mixture of corn, rice, wheat, peas, millet, and sorghum	Starter cultures * Daqu starter: *Mucor racemosus*, *Aspergillus niger*,* Thermomyces lanuginosus*, *Candida* sp., *Saccharomyces cerevisiae*, *Bacillus subtilis*, lactic acid & acetic acid bacteria * Xiaoqu starter: *R*. *oryzae*, *Rhizopus peka*, *S*. *cerevisiae* *Fuqu starter: *Aspergillus* sp., *R*. *oryzae*	China	Liu and Sun 2018
Natto (bean form)	Soybean	*B*. *subtilis*	Japan	Chan et al. 2021
Doenjang (paste form)	Soybean	*Aspergillus sojae* or *A*. *oryzae*	Korea	De Villa et al. 2023
Sufu (bean curd- cheese like product)	Soybean	*Mucor* sp. & *Rhizopus* sp.	China	Cheng et al. 2009
Vinegar	Rice, oat, sorghum, barley, corn, millet, wheat bran, sweet potato	*Aspergillus* sp., *Rhizopus* sp., *Monascus* sp., *S*. *cerevisiae*, *Hansenula anomala*,* Acetobacter* sp.	China	Liu et al. 2004
Ogi (cereal pudding)	Maize, sorghum or millet	*S*. *cerevisiae* & lactic acid bacteria	West Africa	Teniola et al. 2023
Tarhana (powdered soup mix)	Wheat flour	*S*. *cerevisiae* & lactic acid bacteria	Turkey	Degirmecioglu et al. 2016

### Microbial features

Solid-state fermentation systems provide conditions close to the natural habitat of selected microbial strains such as fungi and actinomycetes, implying their terrestrial evolution history (Barrios-Gonzalez 2012). Fungi are the major groups of microorganisms utilized in solid-state fermentation. Especially, the hyphal mode of filamentous fungi (mycelium) enables adaptation to low water activity and osmotic stress conditions, thereby making them superior to unicellular microorganisms. This morphology lets them colonize over the surface of the solid substrate with the ability to penetrate into the substrate to reach the required nutrients (Barrios-González 2012). It is important to note that such types of mycelial morphologies are common for solid-state fermentation systems. The fungal morphology in submerged fermentation can be in different forms: (i) dispersed mycelium, (ii) mycelial clumps, and (iii) dense pellets (Hansen et al. 2015) or combinations of those at the same time. The morphology has a significant effect on the types and amounts of the metabolites produced. Since quite different morphologies can be observed in solid-state and submerged fermentation systems, the microbial metabolites produced in each system can differ to a great extent.

Some of the fungal strains have selectively been well adapted to either solid-state or submerged fermentation conditions (Hansen et al. 2015). For example, Ishida et al. (1998) observed that the glaB gene in *A*. *oryzae* responsible for secretion of the glucoamylase enzyme was markedly expressed under solid-state fermentation conditions, while it was expressed at an extremely low level under submerged bioprocess conditions. Further studies focusing on cloning and expression of the mentioned gene in *Escherichia coli* have proven that its expression is induced by low water activity and high temperature. This explains the high level of glucoamylase secretion by the mentioned strain under solid-state fermentation conditions. In another study, Liu et al. (2020) investigated the enzyme production pattern of *Phanerochaete chrysosporium* under both fermentation conditions. The numbers of identified carbohydrate-active enzymes were almost doubled under solid-state fermentation conditions as compared to the submerged fermentation conditions, indicating the superior potential of the solid-state fermentation. It had been confirmed by the results of several other studies that the solid-state fermentation condition was more effective in the production of higher enzyme titers as compared to submerged fermentation conditions as well (Hansen et al. 2015).

In the case of secondary metabolites, Barrios-Gonzalez et al. (2008) found that solid-state fermentation stimulated the production of lovastatin by *Aspergillus terreus* TUB F-514 due to a higher expression level of the gldB gene under osmotic stress conditions. The expression of the mentioned gene was not observed under submerged fermentation conditions. It is critical to emphasize that sometimes secondary metabolites are produced only under solid-state conditions, although the considered strain can exhibit a very effective growth rate under submerged fermentation conditions. For example, secondary metabolites such as coniosetin and pyrrocidienes A and B are only produced under solid-state bioprocess conditions (Barrios-González 2012). Zhang et al. (2013) also tested the effect of bioprocess mode on pigment and citrinin production by *Monascus purpureus* AS3.531. It has been figured out that the solid-state bioprocess enhanced production of both types of the metabolites while providing a more profound effect on citrinin production, which is a kind of mycotoxin. Amaya-Chantaca et al. (2022) compared the release of phenolic compound amounts from grape pomace by *Aspergillus niger* GH1 under solid-state and submerged bioprocess conditions. Their results implied that solid-state fermentation was a better option due to the release of a higher amount of phenolic compounds.

**Fig. 2:**
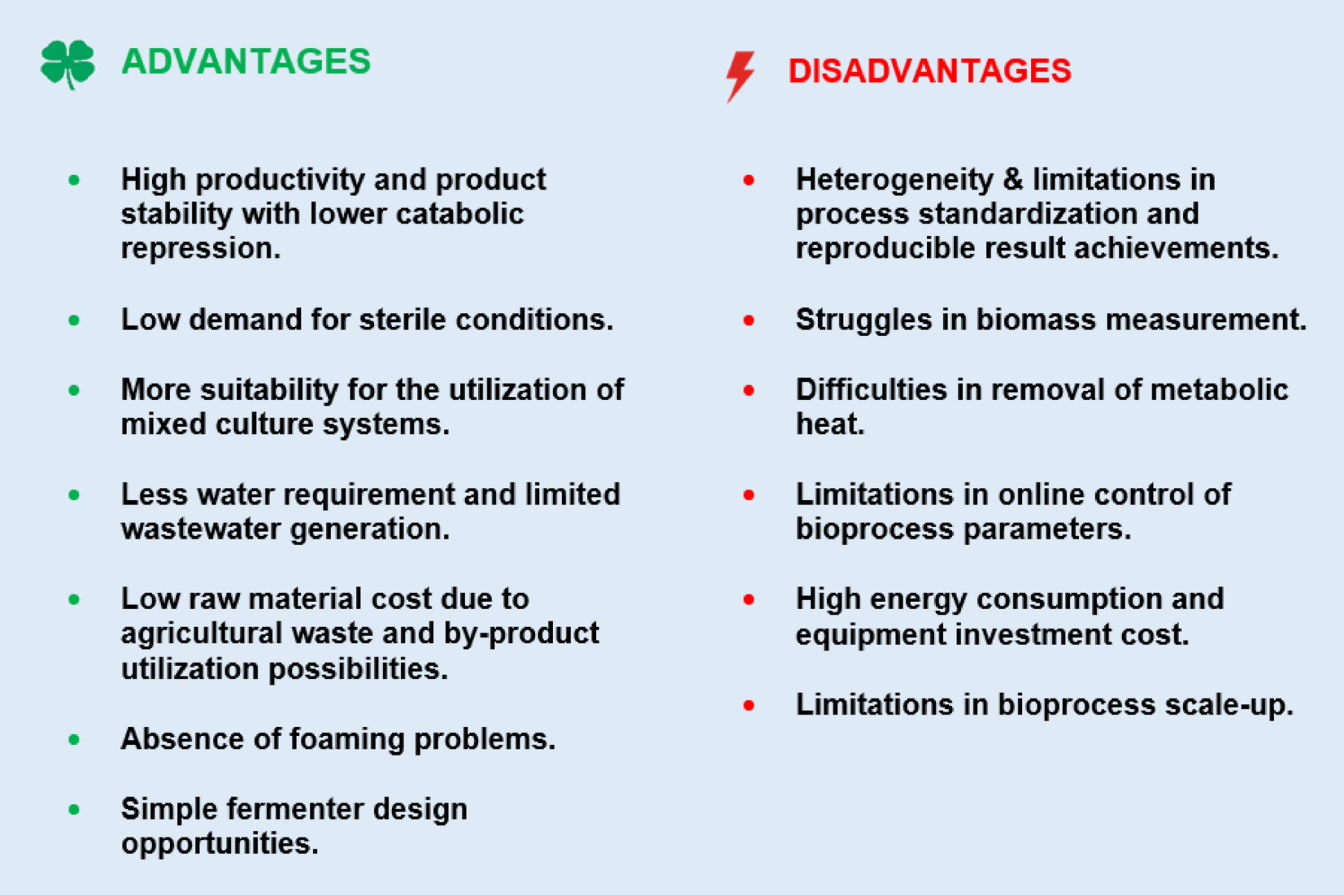
The advantages and disadvantages of the solid-state fermentation system

The results of all the above-summarized studies imply that the activation of some of the genes for secretion of certain metabolites occurs under conditions specific to solid-state fermentation conditions, such as low water activity and osmotic stress conditions. As is proven in various studies, solid-state bioprocess mode is a very powerful bioprocess mode for secretion of high titers of microbial metabolites, especially fungal enzymes and industrially important secondary metabolites. It has additional advantages over submerged bioprocess mode together with certain disadvantages (Singhania et al. 2009; Manan and Webb 2017a), which are summarized in Fig. 2.

### Process variables and cultivation systems

There are various nutritional and bioprocess parameters that have effects on the production of the target microbial metabolite(s). Most of those factors are critical for any kind of bioprocess, either solid-state or submerged, and determination of their optimum levels is of great importance. The common factors for both types of bioprocesses are pH, temperature, aeration, agitation- mixing, moisture content-water activity, inoculum age, and inoculum concentration. However, there are some factors that are much more specific to solid-state fermentation systems with higher importance. The particle size of the substrate is one of these factors. Particle size has an influence on the surface area to volume ratio, which is pivotal for mass and heat transfer phenomena. Smaller particle size implies larger surface area, thereby providing more contact area to microbial strains for accessing the nutrients. Particle size also has an effect on bed porosity, referring to the void volume occupied by air. Although smaller particle size is advantageous because of providing more contact area of nutrient to the microorganisms, a larger surface area can also be advantageous because of providing better respiration/aeration efficiency. It is important to note at this point that a too-small particle size may create substrate agglomeration problems as well. Therefore, the optimum level of the particle size has been clarified in many of the studies considering solid-state microbial metabolite production (Krishna 2005; Singhania et al. 2009; Manan and Webb 2017a; Selo et al. 2021). Another bioprocess parameter that has been mainly tested in solid-state fermentation is the solid-to-liquid ratio. Utilization of excessive water can reduce the bed porosity as a result of filling the pores within the solid materials. Consequently, gas diffusion may get restricted. Excessive water can also promote the formation of bigger pellets. On the other hand, low moisture content can impair the microbial growth due to the mass transfer limitations and poor nutrient solubility possibilities (Zeng et al. 2019; Selo et al. 2021).

**Fig. 3:**
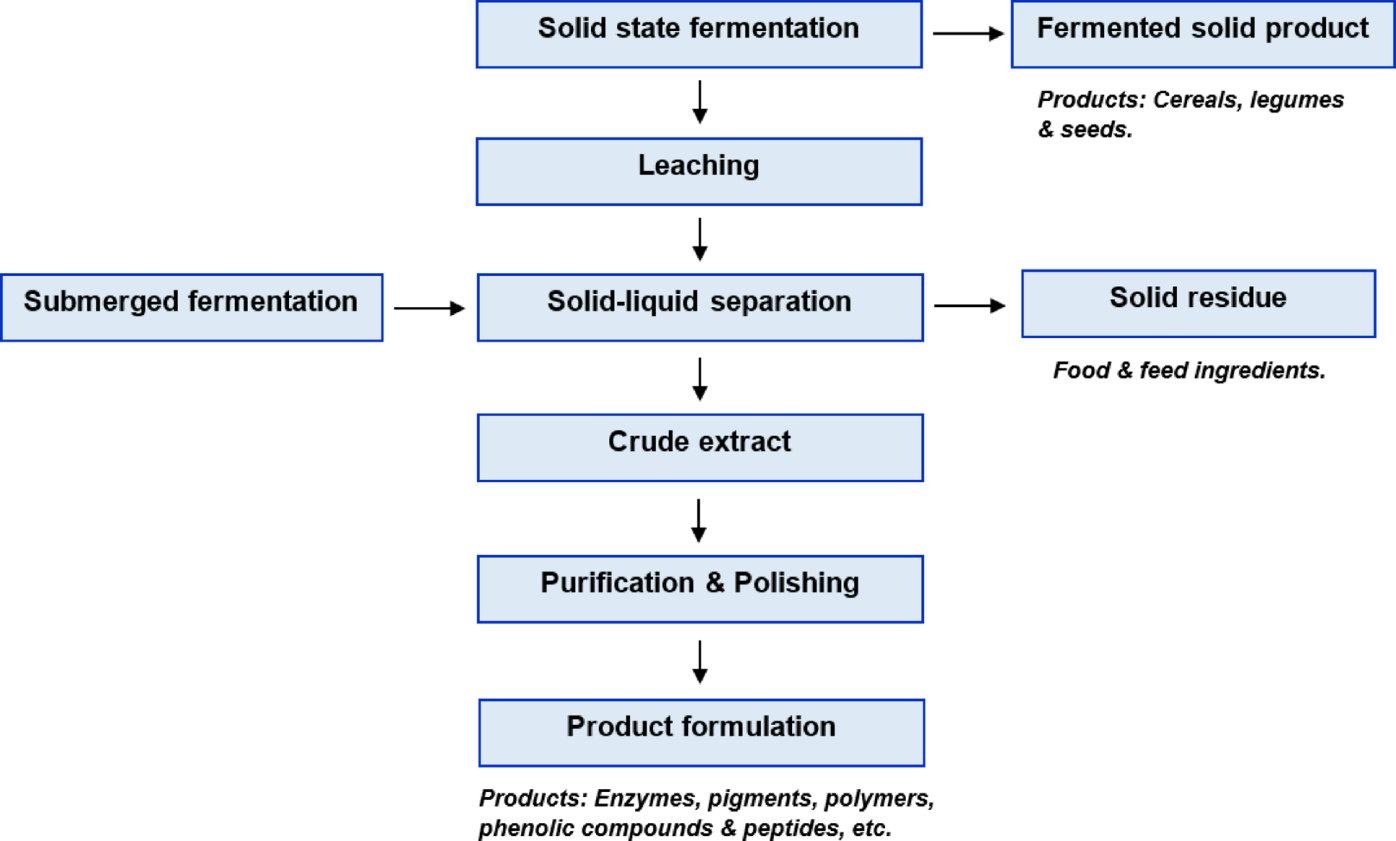
Product recovery and purification operations in solid state and submerged fermentations

There is an extra step at the end of the solid-state fermentation as compared to the submerged fermentation, which is the leaching step, referring to the extraction of the product of interest from the solid material (Fig. 3). Water has generally been used as a leaching solvent in many of the studies. However, depending on the target product, leaching solvents may differ. For example, ethanol- or methanol-based extraction solvents have been used for leaching of the phenolic compounds (Dey and Kuhad 2014; Mao et al. 2020). Some surfactants (e.g., Tween 80) have also been applied comparatively as a leaching solvent in some of the studies, resulting in a higher yield of product recovery (Diaz et al. 2007).

**Fig. 4:**
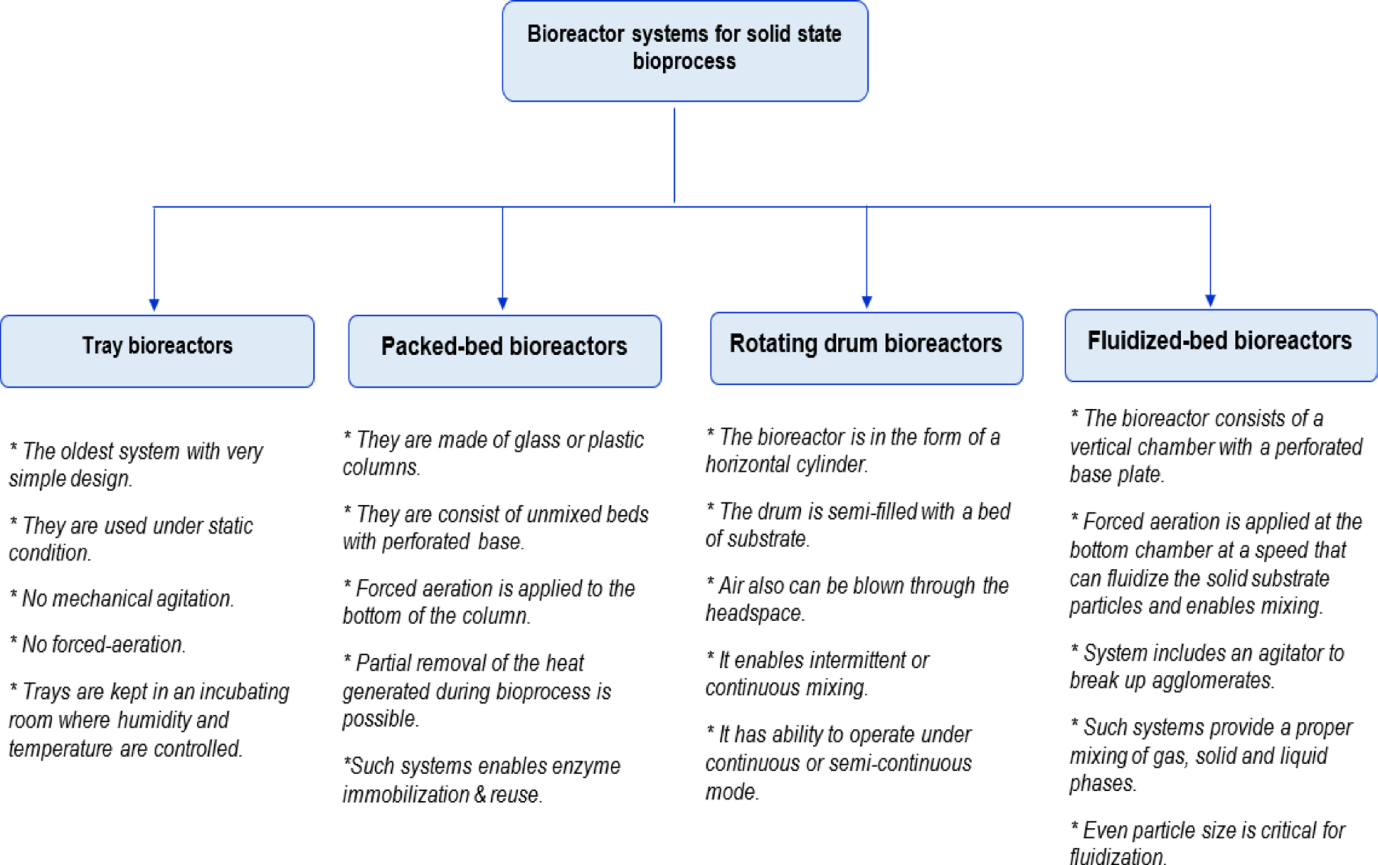
Certain types of bioreactor designs for solid state fermentation systems

There are certain types of bioreactor designs for solid-state fermentation systems: (i) tray bioreactors, (ii) packed-bed bioreactors, (iii) rotating drum bioreactors, and (iv) fluidized-bed bioreactors. Their important aspects are summarized in Fig. 4 (Singhania et al. 2009; Manan and Webb 2017a). The most critical issues in solid-state fermenter systems are removal of excessive heat and supply of sufficient aeration. Therefore, it is important to elucidate the certain requirements of the microbial strain under study for the production of a particular metabolite at a small scale before scaling up the process. Tray bioreactors are the oldest systems with simple designs, operating under static conditions. They are still being mostly preferred for the production of several value-added compounds. Other bioreactor types enabling mixing, such as rotating drum bioreactors, bring the problem of mycelial damage. Therefore, bioreactor choice or design should be carried out considering the target product, the physiological requirements of the microbial strain(s), and the overall bioprocess cost.

Regarding the purification strategies required for the product of interest after solid-state fermentation, it has been mentioned by some of the researchers that the purification process might be easier for the metabolite(s) produced under solid-state fermentation conditions than the submerged fermentation conditions (Manan and Webb 2017a). The basis for this claim was the production of a higher concentration of the metabolite of interest under solid-state fermentation conditions. However, easier purification after solid-state fermentation is not the case in general. Solid-state fermentation results in the simultaneous production of various types of metabolites. For example, production of various types of enzymes occurs when complex substrates are used. The extract obtained after leaching will have so many enzymes and other metabolites at the same time. Downstreaming such a complex extract consisting of various products will need a multi-step operation, thereby increasing the overall bioprocess cost. Even only the crude product recovery by employing leaching at a large scale is an issue in itself. However, there are certain fields (leather industry, detergent industry, etc.) where the high-titer concentrated products (e.g., enzymes) are required rather than the purity degree. Solid-state bioprocess might be more useful in such cases. On the other hand, since the product is being produced in high concentration, a multi-step purification approach may not be a problem if the product is high value with high demand. Therefore, evaluation of the feasibility to a particular purpose is a prerequisite for designing an economical bioprocess.

## Effect of solid-state fermentation on phenolic compound generation

### Structures, biosynthesis, and bioactivities

One of the most important groups of bioactive compounds that is being produced during solid-state fermentation is phenolic compounds. From the structural point of view, the phenolic compounds consist of at least one aromatic ring with one or more attached hydroxyl groups. They may have other functional derivatives such as esters, methyl ethers, and glycosides in their structures. There are several reasons for their biosynthesis in plants. They possess roles in cellular signaling and in processes associated with plant growth and development. They are protective agents as a part of a defense mechanism against biotic (pathogens and herbivores, etc.) and abiotic (radiation, heavy metals, and extreme temperature, etc.) stress factors. They exhibit antimicrobial and antioxidant properties that function in the inhibition of the growth of certain pathogens and alleviation of the undesirable effects of reactive oxygen species, respectively. Moreover, phenolic compounds possess various other bioactivities, such as neuroprotective, antiproliferative, anti-obesity, anti-inflammatory, and anticarcinogenic activities (Albuquerque et al. 2021). However, they have received more attention due to their exceptional level of antioxidant properties, implying their high hydrogen-donating abilities (Roasa et al. 2021). It has been proven that they even exhibit stronger antioxidant properties than the carotenoids, vitamin C, and vitamin E (Dey et al. 2016). Therefore, the majority of the studies carried out regarding the phenolic compounds mainly considered their antioxidant properties. The antioxidant property of phenolic compounds arises from their function in reducing oxidative damage that occurs because of the exposure of excessive reactive oxygen species. Actually, reactive oxygen and nitrogen species are essential in several cellular processes, such as energy supply, immune function, detoxification, and chemical signaling. Under normal circumstances, they are continuously produced in the human body (Dimitrios 2006). However, an imbalance in cellular levels of these reactive species due to their overproduction or exposure to external oxidant substances or failure in defense mechanisms can result in damage to cellular components (e.g., DNA, proteins, and lipids). Consequently, a number of chronic diseases, such as type-2 diabetes, cardiovascular diseases, and certain cancer types, together with aging problems, can be triggered (Roasa et al. 2021; Dimitrios 2006). When phenolic compounds are taken into the body through dietary sources, they can protect the cells against oxidative stress in case of any failure in the inherent antioxidant defense mechanism, and they can strengthen the immune system.

**Fig. 5:**
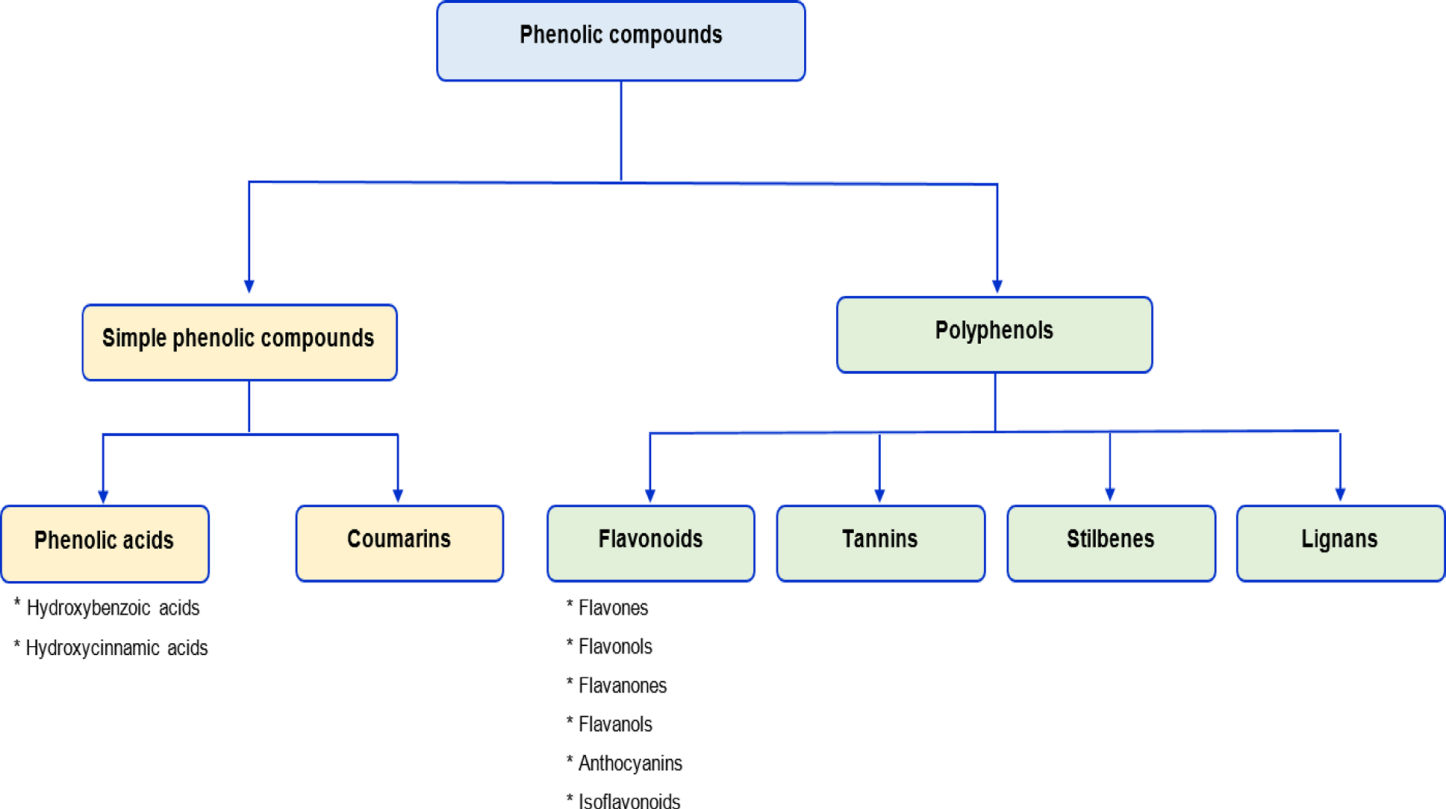
Classification of phenolic compounds

Phenolic compounds that contain one phenol unit or its derivatives are classified as “simple phenolic compounds,” while compounds containing more than one phenol unit are classified as “polyphenols”. Further sub-classifications of both groups are summarized in Fig. 5. Phenolic compounds in plant tissue can be in either soluble or insoluble form. Soluble phenolics can be in “free” forms, or they can be in the form of “conjugates,” which are usually methylated or esterified with low molecular weight compounds such as sugar, glycerol, and spermidine (Li et al. 2022). Insoluble “bound” form phenolics are mainly covalently linked to structural cell wall components such as cellulose, hemicellulose, and pectin as the carbohydrates and the structural proteins. In addition to covalent bonding, hydrophobic interactions and hydrogen bonding can also be seen to some extent (Roasa et al. 2021). The conjugated and bound phenolic compounds can be liberated by acid, alkaline, or enzymatic hydrolysis. A combination of aqueous and organic solvents is commonly employed for the extraction. Generally, the conventional extraction methods based on utilization of aqueous-organic solvents are able to isolate the soluble low-molecular-weight phenolic compounds, while insoluble bound phenolic compounds still remain inside the solid matrix (Li et al. 2022). It has been shown that the bioavailability and health benefits of phenolic compounds may significantly differ depending on their form within the plant matrix. Previous studies indicated that soluble phenolic compounds were more likely to be absorbed into systemic circulation inside the body, pointing out more profound beneficial effects (Roasa et al. 2021). Therefore, approaches aiming at the release of the bound phenolic compounds are of the utmost importance.

### Enhancement of phenolic content by solid-state fermentation

The plant cell wall, where the insoluble bound forms of the phenolic compounds exist, is an extremely complex matrix. The term “lignocellulose” is used for the description of this matrix. It consists of cellulose (40–60%), hemicellulose (20–40%), and lignin (up to 25%) along with small amounts of pectin, protein, extractives, and ash (Andlar et al. 2018; Saini et al. 2022). While cellulose and hemicellulose constitute the main carbohydrate fraction, lignin is a distinct component, classified as a phenolic macromolecule (Andlar et al. 2018; Okonkwo et al. 2023). The antioxidant activity of lignin-based phenolic compounds depends on the functional groups (e.g., phenolic hydroxyl, carbonyl, methoxyl, alkyl, sulfonic acid, and carboxylic acid groups) that they have. It is important to note that the hydroxyl groups (–OH) are the most prevalent group providing the high level of antioxidant capacity to lignin-derived phenolic compounds (Okonkwo et al. 2023). The solid-state fermentation process is one of the important processes that can function in the depolymerization of the lignocellulosic plant cell wall for the liberation of the phenolic compounds, most of which are in an insoluble bound form entrapped within this recalcitrant matrix. At the initial stage of solid-state bioprocess, the microbial strains have very limited access to cellulose and hemicellulose due to their structural connection with lignin. The enzymes synthesized by the microbial cell factories take the main role in the depolymerization of the plant cell wall. Among these enzymes, amylases provide the metabolizable sugar to the cells through hydrolysis of starch. Cellulases (1,4-β-endoglucanase, cellobiohydrolases, and β-glucosidases) (dos Santos et al. 2023) and xylanases (endo-1,4-β-xylanase and β-xylosidase) (Yegin 2023) take roles in the degradation of the cellulose and xylan fractions of hemicellulose, respectively. Complete breakdown of hemicellulose requires synergistic action of various accessory enzymes such as α-glucosiduronase, acetyl xylan esterase, α-l-arabinofuranosidase, ferulic acid esterase, and ρ-coumaric acid esterase (Yegin 2023) since it is a more complex cell wall component than cellulose. All these hydrolase enzymes facilitate the depolymerization of lignin, which is the main source of phenolic compounds. Microbial cell factories are also able to synthesize enzymes (oxidases) that can degrade lignin, which are (i) peroxidase, (ii) manganese peroxidase, (iii) versatile peroxidase, (iv) dye-decolorizing peroxidase, and (v) laccase (Weng et al. 2021). Among the microbial strains, fungi have been indicated as the most potent strains in lignin modification/degradation because of possessing a wide range of lignin-degrading enzyme systems (Andlar et al. 2018). This point also reveals the usefulness of solid-state fermentation for bioactive phenolic compound production, as this bioprocess mode is more suited to the natural habitat of the fungi, as indicated before. Especially the white-rot fungi, including basidiomycetes and ascomycetes, are well-known fungal strains with lignin degradation ability in nature. *Phanerochaete chrysosporium* has been widely applied in lignocellulosic biomass pretreatment (Weng et al. 2021). Recent studies revealed that some bacteria (e.g., *Actinobacteria*) also exhibited lignin-degradation abilities (Weng et al. 2021). It is important to emphasize that solid-state bioprocess can be applied both for the production of valuable lignin-derived fine chemical compounds with bioactivities and also for the purpose of lignin removal/modification as a pretreatment step.

Solid-state fermentation has been applied for the enhancement of the phenolic content of cereals and legumes or the recovery of phenolics from agro-industrial wastes and by-products. In most of the studies, the total phenolic content and antioxidant capacity have been used as an indication. However, identification and quantification of specific groups of phenolic compounds has also recently received attention (Dulf et al. 2023; Selo et al. 2024; Nemes et al. 2025).

### Microbial strains used in solid-state phenolic compound generation

Fungi from *Aspergillus* sp. and *Rhizopus* sp. and lactic acid bacteria are commonly employed in bioprocesses targeting production of phenolic compounds by solid-state fermentation due to their GRAS status (Generally Recognized as Safe). The bakery yeast, *Saccharomyces cerevisiae*, is also used in various studies because of being safe. These strains are especially utilized in upgrading the nutritional value of cereals and legumes from the phenolic content point of view (Wu et al. 2022; Suprayogi et al. 2022; Purewal et al. 2024). In some cases, spontaneous fermentation has been preferred (Sobowale et al. 2024). However, it is worthy of emphasizing that figuring out the relationships between the microbial physiology and the phenolic compound production is critical for the development of efficient bioprocess strategies. From this perspective, recent studies utilized different strains, such as *Trametes versicolor* TV6 (Selo et al. 2024) and *Actinomucor elegans* ATCC 22963 (Dulf et al. 2023,) for solid-state phenolic compound production from different fruit pomaces. *T*. *versicolor* sp. are well-known phenolic compound producers due to their lignin-degrading enzyme systems (e.g., laccase) (Tisma et al. 2021). In the last couple of years, researchers also paid more attention to the utilization of co-culture systems in solid-state phenolic compound production (Olukomaiya et al. 2020; Wu et al. 2022; Hu et al. 2022) in order to utilize the enzyme systems of different microorganisms simultaneously. There are also studies focusing on sequential utilization of different microbial strains. For example, Suprayogi et al. (2022) utilized *Bacillus subtilis and A*. *oryzae* sequentially in solid-state fermentation of soybean. The very recently performed selective studies on solid-state phenolic compound production from various cereals, legumes, and agro-industrial residues are summarized in Table 2. Elucidation of the kinetics of phenolic compound release during solid-state bioprocess is of critical importance since at certain times of the bioprocess, it is possible to come across the degradation and/or biotransformation of the target compound(s) to other compounds. For example, in a study performed by Paz-Arteaga et al. (2023), the total phenolic compound release and antioxidant activity have changed during solid-state fermentation of pineapple waste by *A*. *niger* GH1. It has been indicated that the reduction might be as a result of decomposition or consumption of certain phenolic compounds by the strain during the exponential phase. In general, phenolic compounds such as quercetin, syringic acid, gallocatechin, and protocatechial acid were said to exhibit a reduction trend after being released during the prolonged fermentation (Paz-Arteaga et al. 2023).

**Table 2:** Influence of solid state fermentation on bioactive phenolic compounds of cereals, legumes and agro-industrial residues

Substrate group		Microorganism	Influence	Reference
Cereals	^a^ Wheat bran	*Aspergillus niger* ATCC-6275	Increase in: TPC, antioxidant activity, various types of specific phenolic compounds (e.g., vanillic acid, ferulic acid).	Nemes et al. 2025
	Wheat bran	*Bacillus* sp. TMF–2	^d^ Increase in: TPC, antioxidant activity Reduction in: phytic acid	Tanaskovic et al. 2021
	Wheat bran	*Enterococcus faecalis* M2	Increase in: TPC, antioxidant activity, gallic acid, syringic acid, ρ-coumaric acid, ferulic acid, cinnamic acid. Reduction in: ρ-hydroxybenzoic acid, chlorogenic acid No change in: phytic acid	Mao et al. 2020
	Whole grain oat	^*b*^ *Rhizopus oryzae &* *Lactobacillus plantarum B1-6*	Increase in: TPC, antioxidant activity	Wu et al. 2022
	Barley	*Aspergillus awamori* MTCC-548	Increase in: TPC, antioxidant activity, tannin	Purewal et al. 2024
	^a^ Black rice	*Aspergillus oryzae* CCTCC M 2023533	Increase in: TPC, FC, antioxidant activity Variation in: specific types of phenolic acids (e.g., vanillic acid & syringic acid)	Zhong et al. 2025
Legumes	Soybean	^*c*^ *Bacillus subtilis* *Aspergillus oryzae*	Increase in: TPC, antioxidant activity Reduction in: tannin, phytic acid, saponin	Suprayogi et al. 2022
	Faba bean	*Bacillus pumilus* CICC 10440	Increase in: TPC, FC, antioxidant activity Reduction in: phytic acid, proanthocyanidin	Xu et al. 2023
	Pigeon pea flour	Spontaneous fermentation	No change in: TPC Reduction in: FC, phytate Increase in: tannin	Sobowale et al. 2024
	Chickpea	*Pediococcus pentosaceus*	No change in: caffeic acid, chlorogenic acid Increase in: TPC, FC, gallic acid, syringic acid, ρ-hydroxybenzoic acid, protocatechuic acid, vanillic acid Reduction in: phytic acid, ferulic acid, ρ-coumaric	Zhou et al. 2025
	Lupin	^*b*^ *Aspergillus sojae &* *Aspergillus ficuum*	Increase in: TPC Reduction in: phytic acid	Olukomaiya et al. 2020
Fruits & Vegetables	Apple pomace	*Actinomucor elegans* ATCC 22963	^d^ Increase in: TPC, antioxidant activity, various types of individual phenolic substance (e.g. chlorogenic acid)	Dulf et al. 2023
	Citrus pomace	^b^ *Lactobacillus plantarum* P10 & *Bacillus subtilis* BF2	Increase in: TPC, naringin, hesperidin	Hu et al. 2022
	Pineapple waste	*Aspergillus niger* GH1	^d^ Increase in: TPC, antioxidant activity	Paz-Arteaga et al. 2023
	Grape pomace	*Trametes versicolor* TV6	Reduction in: TPC, FC, antioxidant activity Variation in: concentration of specific phenolic compounds	Selo et al. 2024

^a^ Pretreatments prior to fermentation have been applied

^b^ Co-culture systems

^c^ Two-steps (sequential) solid state fermentation

^d^ Concentration changes (increase or decrease) depending on the fermentation time as compared to initial stage

TPC: Total phenolic content, FC: Flavonoid content

## Effect of solid-state fermentation on nitrogenous bioactive compound generation

### Plant proteins

In addition to the lignocellulosic fraction, the protein fraction is another important plant constituent that the solid-state fermentation has an influence on. There is already a growing interest in plant-based proteins even if we do not consider the solid-state fermentation. The main reason for this growing interest is the sustainable production pattern of the plant-based proteins as compared to the animal-based proteins. Although animal-based products are important dietary protein sources (22–27%) with the benefit of providing optimum levels of essential amino acids, their production is not sustainable when we consider the high growth rate of the world population and the environmental issues that appear as a result of their production (Canoy et al. 2024). Personal preference for vegan and vegetarian nutritional models and lactose intolerance are the other reasons for the increased demand for plant-based proteins.

Development of meat alternatives by utilization of plant-based proteins is a challenging research field due to several reasons. The protein content of the plants is much lower as compared to the animal-based resources. They lack some varieties of essential amino acids. They have additional disadvantages such as limited digestibility and the presence of anti-nutritional factors (e.g., tannin and trypsin inhibitors) (Farid et al. 2024). Solid-state fermentation is a very useful tool to overcome such limitations. Structural modification of plant-based materials by solid-state bioprocess enables (i) enhancement of protein digestibility, (ii) hydrolysis of polypeptides, (iii) generation of peptides with bioactive properties, (iv) modification of amino acid profile, and (v) elimination of anti-nutritional factors (Feng et al. 2024).

### Modification of plant proteins by solid state fermentation

In a solid-state bioprocess, microbial strain(s) consume the protein as a nitrogen source at the initial stage. As a result of microbial metabolism, secretion of several metabolites occurs. Protease enzymes are the main group of enzymes that take the role in plant protein modification. Proteases are categorized into six subclasses according to their catalytic mechanisms: cysteine proteases, serine proteases, aspartic proteases, threonine proteases, metalloproteases, and proteases of unknown type (Yegin et al. 2011). It is important to know the catalytic mechanism of the proteases that are being secreted by the microorganisms during a fermentation. Based on this knowledge, the solid substrate can be chosen. From another perspective, depending on the protein profile of the solid substrate under study, the microbial strain(s) secreting the substrate-compatible proteases can be selected. Strains producing different types of proteases can be used as co-culture systems, or they can be used sequentially for achieving an optimum level of hydrolysis.

During solid-state fermentation, in addition to proteases, the other enzyme systems (e.g., lignocellulosic enzymes) also facilitate the hydrolysis of proteins by degrading the recalcitrant plant cell wall materials. Just like various other enzyme systems, fungal strains are the major microbial cell factories for secretion of the proteases. In general, fungi possess a wider range of protease enzymes as compared to bacteria, although lactic acid bacteria exhibit great potential in the release of peptides with bioactive properties (Green et al. 2024). The proteolytic enzymes produced during solid-state fermentation hydrolyze the macromolecular proteins into smaller protein fragments, peptides, and amino acids. Consequently, the formation of various fragments of different molecular weights occurs. These different sizes of small fragments are important from several aspects. The released peptides may have bioactivities such as antioxidant and antihypertensive activities. Reduction in molecular size implies increased protein digestibility since peptides of small size and free amino acids can easily be absorbed by the gastrointestinal system in the body. Moreover, improved nutritional value and enhanced flavor are other significant achievements accompanied by the action of proteases during solid-state fermentation.

#### Effect of solid-state fermentation on protein digestibility

Protein digestibility refers to the extent to which a protein can be hydrolyzed by proteolytic enzymes. Protein digestibility is influenced by several factors, including the structural characteristics of the protein, the intensity of thermal processing, and the presence of certain compounds known as anti-nutritional factors, which can adversely affect protein digestion (Terefe et al. 2021). Regarding proteolytic hydrolysis-based processes, it is noteworthy to define the term “degree of hydrolysis” at this point. It is accepted as a key parameter for monitoring protein hydrolysis. It can be defined as the percentage of peptide bonds that have been cleaved within the total peptide bonds (Nielsen et al. 2001). Several methods are being used for determination of this parameter, such as trinitrobenzenesulphonic acid (TNBS) and o-phthalaldehyde (OPA) methods based on amino acid determination (Feng et al. 2024). Osmometry, pH-stat, and soluble nitrogen content are other methods that have also been used for determination of the same parameter (Nielsen et al. 2001).

The term “degree of hydrolysis” has often been utilized as a major parameter in studies focusing on solid-state upgrading of the nutritional status of many grains and legumes. In a study performed by Chin et al. (2022), brewer’s spent grain was fermented by *Rhizopus oligosporus* ATCC 64063, and the results revealed a hydrolysis degree of 14.6% by the TNBS method. The researchers emphasized that the hydrolysis degree that they obtained by solid-state fermentation of the same substrate was higher than the maximum hydrolysis degree (13%) obtained by commercial enzyme preparation in another study performed by Celus et al. (2007). However, the fermentation period (3 days) was longer than the enzymatic hydrolysis period (2 h). In another study (Wu et al. 2018), whole grain oats were fermented by *R*. *oryzae* and *L*. *plantarum* B1-6 individually and also by their co-culture system. The results of this study have shown the usefulness of co-culture systems in achieving a higher degree of hydrolysis as compared to single-culture utilization. Ayyash et al. (2019) performed a detailed study employing flours of 3 different substrates separately (white quinoa, wheat, and sweet lupine) by utilizing 3 different lactic acid bacteria as a single-culture system. Among the tested substrates and lactic acid bacteria varieties, the highest increase in hydrolysis degree as compared to the specific control of each substrate was obtained for lupine flour when fermentation was carried out by *Lactobacillus reuteri* KX88177 (from ∼8.5 to 35%). This strain revealed the highest degree of hydrolysis over other strains utilized in the study, independent of the chosen substrate type. The higher protein content of lupine and the better performance of the endogenous lupine proteolytic enzymes could be the possible reasons for the highest degree of proteolysis obtained for this substrate. Moreover, the protein pattern of the lupine flour might be more compatible with the proteolytic enzymes of this lactic acid bacterium.

Another important term regarding proteolytic hydrolysis, which has been frequently utilized in recent studies, is “in vitro protein digestibility- IVPD”. It refers to the degree of hydrolysis of proteins into smaller peptides and amino acids in a controlled laboratory setting that mimics the digestive tract of the human body to some extent. The human digestive system is responsible for the breakdown of the foodstuff by mechanical, chemical, and enzymatic actions, thereby releasing the nutrients in a form that the cells can utilize (Orlien et al. 2023). Since it is obviously not possible to withdraw samples from each part of the human digestive system, in vitro systems are commonly employed to understand the changes in dietary proteins, their functionalities, and bioavailabilities after digestion.

Solid-state fermentation significantly impacts protein fractions; therefore, IVPD has been employed to clarify the effects of fermentation on the digestibility of proteins. In general, fermentation is a suggested process for the improvement of IVPD of plant-based proteins. However, the influence of fermentation exhibited variations on this value. In a study performed by Chen et al. (2017), solid-state fermentation by A. *oryzae* CICC 2436 was utilized as a strategy to improve the bioactive properties of the soybean protein isolate. This study demonstrated the usefulness of the fermentation by conversion of isoflavone glycosides into aglycones and glyceollins, which were kept in the protein isolate as a result of the interaction between proteins and polyphenols, thereby enriching the bioactive properties of the protein isolate. The fermented protein isolate exhibited higher solubility and in vitro digestibility than the unfermented counterpart. Similarly, Asensio-Grau et al. (2020) showed that the solid-state fermentation of lentil flour by *Pleurotus ostreatus* was a useful approach for increasing the in vitro protein digestibility of the mentioned flour. In a very informative study performed by Chandra-Hioe et al. (2016), the effects of fermentation on trypsin inhibitor activity and in vitro protein digestibility of different legumes (faba bean, desi chickpea, and kabuli chickpea) were elucidated. The fermentations were carried out by employing commercial yogurt cultures because of being a simple and viable technique. The trypsin inhibitor activity decreased by 2.7%, 1.1%, and 4.7% for desi chickpea, kabuli chickpea, and faba bean, respectively. The in vitro protein digestibility of all the samples increased while trypsin inhibitor activity reduced simultaneously. The results of this study indicated that the reduction in anti-nutritional factors (trypsin inhibitors) could be linked to the achievement of higher in vitro protein digestibility.

Some lactic acid bacteria varieties, such as *L*. *plantarum* B1-6, were also found as effective strains for enhancing the in vitro protein digestibility of soy seeds (Rui et al. 2017). Comparative studies to elucidate the effects of single- or co-culture systems on in vitro protein digestibility have also been performed. In vitro protein digestibility of maize flour was improved by 40%, 36%, and 34% for *L*. *plantarum*, *Saccharomyces cerevisiae*, and their co-culture, respectively. In this study, the proteolytic enzyme profile of the lactic acid bacteria has been thought of as a possible key factor leading to better digestibility (Terefe et al. 2021).

Summarizing, the possible mechanisms for the improvement of the in vitro protein digestibility by fermentation can be linked to: (i) reduction in anti-nutritional factors that have the potential to inhibit digestive enzymes (e.g., trypsin and chymotrypsin inhibitors), (ii) protein cross-linking with phenolic compounds, and (iii) secretion of microbial proteases during solid-state fermentation that take the role in partial degradation of the proteins.

There are also studies that resulted in reduced in vitro protein digestibility after solid-state fermentation. For example, solid-state fermentation of de-oiled rice bran by *R*. *oryzae* NCIM 1009 led to a reduction of 16.5% in protein digestibility (Ranjan et al. 2019). Olukomaiya et al. (2020) utilized *A*. *sojae* ATCC 9362 and *Aspergillus ficuum* ATCC 66876 comparatively as single-culture and co-culture systems in the fermentation of lupine flour. After fermentation, the initial in vitro protein digestibility (44.74%) of the unfermented lupine flour reduced to 34.25%, 37.58%, and 30.22% for *A*. *sojae*, *A*. *ficuum*, and their co-culture, respectively. The reduction in protein digestibility of the fermented flours may be attributed to the entrapment of protein within the fiber matrix due to insufficient degradation/modification of the substrate during fermentation. The microbial strain(s) may lack some enzyme systems, or the fermentation conditions may not be proper for their secretions to effectively solubilize the fiber matrix. Furthermore, the partial denaturation of proteins during the drying process may contribute to a reduction in both protein dispersibility and solubility, consequently leading to a decrease in the in vitro protein digestibility (Olukomaiya et al. 2020).

#### Effect of solid-state fermentation on bioactive peptide generation

Solid-state fermentation has been specifically used for the production and identification of novel peptides exhibiting specific bioactivities. Bioactive peptides are chains of amino acids ranging from 2 to 20 residues (less than 6000 Da) exhibiting biological activities such as antioxidant, antihypertensive, hypocholesterolemic, or immunomodulatory (Canoy et al. 2024). These peptides are embedded within the parent protein in inactive forms. Biological functions can be exhibited upon the release of these peptides, which require specific actions of protease enzymes. Bioactive peptides also gained attention because of their potential to extend the shelf life of food products by replacing synthetic preservatives (e.g., butylated hydroxytoluene-BHT and butylated hydroxyanisole-BHA) possessing certain health risks (Farid et al. 2024).

Bioactive peptides are commonly produced by the utilization of various types of commercial protease preparations such as papain, bromelain, pepsin, trypsin, and chymotrypsin. They can be produced by chemical synthesis or chemical extraction as well. Enzymatic hydrolysis and microbial fermentation are the preferred methods employed in commercial production (Wang and Ma 2024). Especially, microbial fermentation by certain lactic acid bacteria became a very attractive approach, as fermentation brings various advantages in addition to peptide release. Generally, bacteria have been accepted as the more potent group of microorganisms for bioactive peptide production as compared to fungi, although fungi possess a wide range of proteases (Wang et al. 2023; Green et al. 2024). It is important to note that production of bioactive peptides and amino acids can also be possible through some intrinsic microbial metabolic pathways in addition to enzymatic modification during fermentation (Green et al. 2024). Bacteria such as *Bacillus subtilis* and *Lactobacillus* sp. are frequently employed strains for the production of peptides possessing antioxidant activity (Lorenzo et al. 2018).

The amino acids that exhibit the highest reactivity include those containing sulfur, specifically cysteine and methionine, as well as the aromatic amino acids phenylalanine, tyrosine, and tryptophan, and also histidine, which contains an imidazole ring. Peptide sequences having such types of reactive amino acid residues exhibit enhanced antioxidant potentials (Farid et al. 2024). The molecular weights of the peptides also have influence on their antioxidant property. In a study performed by He et al. (2012), rapeseed meal was fermented by *B*. *subtilis* 10160. Peptides of different molecular weights (180–5500 Da) were obtained. The peptide of lower molecular weight exhibited the higher radical scavenging activity. The same researcher also emphasized the importance of fermentation time on the molecular weight of the peptides obtained. During prolonged fermentation, the amount of peptides with smaller molecular weight and the radical scavenging activity were simultaneously decreased. The consumption of the small peptides by the microbial strain might be the possible explanation for this result.

Among the biological activities that the peptides produced by fermentation possess, the metal-chelating activity recently received more attention. It has been indicated that such kinds of peptides having the chelating ability through covalent bonding have positive impacts on several body functions due to the properties accompanied by chelating, such as antihypertensive, antithrombotic, and hypolipidemic (Li et al. 2023). It is important to note that angiotensin-converting enzyme (ACE)-inhibitory activity is one of the critical antihypertensive activities that received a special emphasis. Regarding the fermentative production of metal-chelating peptides, an interesting study was performed by Li et al. (2023) using *A*. *oryzae* and soybean meal. Among the various peptide fractions of different molecular weights, the peptides smaller than 20 kDa exhibited stronger chelating capacity than the other fractions. The chelating capacities of all peptides of different fractions were significantly higher with Zn^2+^ than with Cu^2+^. In another study performed by Wang et al. (2022), solid-state fermentation of rapeseed meal was carried out by co-culture of *B*. *subtilis* YY-4 and *L*. *plantarum* CICC6026. After fermentation of the substrate, the concentration of small peptides (< 1 kD) was significantly increased together with antioxidant and immunomodulatory activities. Interestingly, no significant change was observed in total phenolic content, implying the generation of peptides with antioxidant and immunomodulatory activities.

There are also studies that utilized solid-state fermentation as a tool for mass production of peptides where the bioactivities that they might have were not considered (Wang and Ma 2024). In some of the studies, different types of bioactivities were observed after solid-state fermentation. However, such bioactivities may or may not be because of the generated peptide itself. For example, Starzynska-Janiszewska et al. (2020) studied the effect of fermentation on some properties of pumpkin oil cake (without hull) by *Rhizopus oligosporus* ATCC 64063. Improvements such as a high level of peptide generation, a reduction in phytate content, an increase in total phenolic content, and antioxidant capacity were obtained. The higher antioxidant capacity of the fermented substrate might be as a result of different fermentation-generated compounds, such as phenolics, in addition to the peptides.

Some of the studies considering the production of bioactive peptides by solid-state fermentation of agricultural materials are summarized in Table 3. It can be clearly observed after checking the previous studies that *Bacillus* sp. are the primary microorganisms utilized in the production of bioactive peptides via solid-state fermentation (Canoy et al. 2024). Since *Bacillus* sp. are known as extraordinary producers of protease enzymes (Raphel and Halami 2024), their utilization in peptide production is a logical strategy. Among the substrates, soybean seems the most commonly preferred substrate for fermentative bioactive peptide production. The main reason for utilization of soybean and by-products of its processing should be the high protein content (~ 40–50%) of this substrate (Lu et al. 2025). However, it also contains some anti-nutritional factors and allergenic proteins (ß-conglycinin and glycinin) hindering its digestion (Li et al. 2023). The potential of solid-state fermentation to eliminate these drawbacks is the other possible reason for the higher preference for this substrate.

**Table 3:** Solid state fermentation of various agricultural materials for production of bioactive peptides

Substrate	Microorganism	Peptide properties	Reference
Soybean meal	*Streptomyces* sp. SCUT-3-3940	Antihypertensive and antioxidant MW < 2000 Da	Lu et al. 2025
Soybean meal	*A*. *oryzae*	Metal chelating peptide	Li et al. 2023
Soybean meal	*B. subtilis* SBM_1	MW < 3000 Da	Liu et al. 2024
Cottonseed meal	*B*. *subtilis* BJ-1	Antioxidant MW < 1000 Da	Sun et al. 2015
Corn gluten meal	*B*. *subtilis* MTCC5480	^a^ Antioxidant	Jiang et al. 2020
Walnut protein meal	*B. subtilis GIM 1.135*	Antioxidant and Fe^2+^ ion-chelating activity	Wu et al. 2014
Rice bran	*B. subtilis* (natto)	Antioxidant	Bisly et al. 2022
Rice bean	*B*. *subtilis* KN2B	ACE-inhibitory	Padhi et al. 2022
Rapeseed	*B*. *subtilis* 10160	Antioxidant MW: 180–3000 Da	He et al. 2012
Rapeseed meal	*B*. *subtilis* YY-4 and *L*. *plantarum* CICC6026	Antioxidant and immunomodulatory	Wang et al. 2022
Sesame meal	*L*. *plantarum* and *B*. *subtilis*	^b^ Antioxidant	Fazhi et al. 2014
Kenaf seed	*Lactobacillus casei* ATCC334	Antibacterial	Arulrajah et al. 2020
Tartary buckwheat	^*c*^ *Lactiplantibacillus plantarum* ATCC 14917	ND	Wang and Ma 2024
Pumpkin oil cake (without hull)	*Rhizopus oligosporus* ATCC 64063	^d^ Antioxidant	Starzynska-Janiszewska et al. 2020
Brewer’s spent grain and soybean meal mixture	*Bacillus licheniformis* CPB2	MW 1000–3000 Da increased MW < 500 Da decreased	Liu et al. 2023

^*a*^ In vivo antioxidant property

^*b*^ In vivo and in vitro antioxidant properties

^*c*^ The strain has been screened as the best peptide producer among tested lactic acid bacteria

^d^ Antioxidant activity might be because of peptides and/or other fermentation produced products such as phenolics

ND: Not determined.

#### Effect of solid-state fermentation on amino acid content

Solid-state fermentation also has a significant effect on amino acid content. In general, the free amino acid content increased due to the solid-state fermentation (Heo et al. 2020; Wang et al. 2022; Liu et al. 2024; Feng et al. 2024). The accumulation of amino acids can be a result of not only hydrolysis by proteolytic enzymes but also microbial metabolism through specific metabolic pathways. As indicated before, modification of the amino acid profile is a crucial issue for plant-based proteins since they lack certain types of essential amino acids. In order to overcome this limitation, a combination of different substrates having complementary amino acid patterns may be used in solid-state fermentation. For example, Heo et al. (2020) utilized mixed grain (wheat germ, wheat bran, oats, barley, brown rice, lentils, and quinoa) in solid-state fermentation by *Bacillus amyloliquefaciens* 245. The researchers obtained an increase in essential amino acid contents during the time course of the fermentation. Similarly, Liu et al. (2023) used a mixture of soybean meal and brewer’s spent grain in solid-state fermentation by thermophilic *Bacillus licheniformis* CPB2 under non-sterile conditions. At the end of fermentation, the content of all types of free amino acids increased except arginine and cysteine. An important point that should be acknowledged is that the prolonged fermentations may result in the consumption of the free amino acids produced, depending on the microbial strain utilized. Fungal strains have also been utilized to obtain a balanced amino acid profile in addition to bacterial strains. In a study performed by Chen et al. (2013), soybean meal was fermented by *A*. *oryzae* ATCC 12892, which resulted in a significant increase in total amino acid content.

## Potential of solid-state fermentation on reduction of anti-nutritional factors

There is another critical issue that needs to be emphasized while considering solid-state fermentation of plant-based resources. During solid-state fermentation, it is possible to observe reductions in the concentration of some of the compounds that are accepted as anti-nutritional factors, especially in cereals, legumes, and their by-products. These compounds take roles in the reduction of nutrient absorption, mineral bioavailability, and protein digestibility (Samtiya et al. 2020; Xu et al. 2023). The commonly studied major anti-nutritional factors are phytic acid and tannins during solid-state fermentation (Xu et al. 2023).

Phytate (inositol hexakisphosphate) is the salt form of phytic acid that exists in plants and is an organic form of phosphorus (60–90% of total phosphorus concentration) (Thakur et al. 2019; Feizollahi et al. 2021). Utilization of microbial strains producing phytase enzyme is a way of decreasing the phytic acid content in plants, since this enzyme catalyzes the hydrolysis of phytic acid to inorganic phosphate and myo-inositolphosphate derivative (Feizollahi et al. 2021; Tanaskovic et al. 2021). In various studies considering solid-state upgrading of legumes, phytic acid content has been taken into consideration as a major anti-nutritional compound (Olukomaiya et al. 2020; Suprayogi et al. 2022; Zhou et al. 2025).

Tannins are another group of antinutritional factors. They are heat-stable polyphenolic compounds, and their antinutritional property mainly results from their role in the reduction of protein digestibility (Thakur et al. 2019). There are two types of tannins: condensed tannins (proanthocyanidins) and hydrolyzable tannins (Purewal et al. 2024; Thakur et al. 2019). The amount of condensed tannins is generally higher in plant materials. Similarly, utilization of microbial strains having tannase enzyme systems, which are able to degrade tannins, can be efficiently used for the removal of tannins. For example, in a study performed by Espitia-Hernandez et al. (2022), A. *oryzae* and A. *nige*r Aa210 have been comparatively used in solid-state fermentation of sorghum. The findings demonstrated that *A*. *oryzae* resulted in reduced tannin content, while *A*. *nige*r Aa210 led to an increase in tannin content. *A*. *oryzae* is known as a strain capable of producing tannase. Therefore, the results of the mentioned study are in line with the previous knowledge (Mizuno et al. 2014).

Enzyme inhibitors are also accepted as an important group of anti-nutritional factors. Protease and amylase inhibitors are the main group of enzyme inhibitors that are present in plant-based resources. They hinder enzyme activity via the catalytic mechanism by blocking the active site of the enzymes (Samtiya et al. 2020). Among these inhibitors, trypsin inhibitors are major inhibitors that have been specifically studied. Trypsin inhibitors are substances that obstruct protein digestion by impeding the function of trypsin. Various studies have proven the potential of solid-state fermentation to decrease trypsin inhibitor levels, thereby increasing the protein digestibility. Trypsin inhibitor activities from bacteria (e.g., *Bacillus* sp.), fungi (e.g., *Aspergillus* sp.), and baker’s yeast during solid-state fermentation have been reported (Feng et al. 2024).

## Biorefinery and zero-waste perspective

It is a prerequisite to transform the petroleum-based production systems to the bio-based production system for the establishment of a sustainable economy. Efficient utilization of renewable resources for the manufacturing of a spectrum of value-added products such as food ingredients, platform chemicals, biopolymers, and biofuels with minimum waste generation is the key for opening a new era from the point of sustainability. This key actually refers to the term: “biorefinery.”

Agricultural biomass possesses a significant potential as a renewable resource. Agricultural wastes and by-products, particularly those that do not compete with the food supply chain, are of primary interest for manufacturing products with high market value (Yegin et al. 2019). Especially, simultaneous production of multiple high-value products using different fractions of such biomass is critical from the zero-waste perspective. Solid-state fermentation-based processes exhibit exceptional potential for this purpose. It is already well known that solid-state fermentation enables simultaneous secretion of various enzymes at high titers targeting the different fractions of the agro-based biomass. Although solid-state bioprocess can enable production of various other types of microbial products, enzymes are the prominent products, first coming to mind among others. There are currently several companies actively using solid-state fermentation for the manufacturing of several products. For example, Biocon in India (Manan and Webb 2017b), Takabio in Japan, and Alltech in Mexico are companies using solid-state fermentation for enzyme production. BOC Sciences in the USA produces a wider range of bioproducts by utilizing solid-state fermentation, such as terpenes, organic acids, peptides, amino acids, phenolic compounds, pigments, aroma compounds, and biosorbents. In addition to direct production of specific microbial metabolites, solid-state fermentation has been typically used as a saccharification step to provide fermentable sugars, which could be easily used by microbial strains for further production of various commercially significant metabolites such as bioethanol. Saccharification of the biomass can also be performed by utilization of different types of commercial enzyme preparations. However, as indicated before, utilization of these enzyme preparations will increase the overall bioprocess cost to a great extent. Solid-state fermentation offers a significant opportunity to decrease such extra costs because of providing simultaneous production of various enzymes. Another issue that should be emphasized regarding the effectiveness of solid-state fermentation in processes designed based on saccharification of the lignocellulosic biomass is that the biosynthesis of several enzymes can be stimulated by high sugar concentration in solid-state fermentation, while this much high sugar concentration generally exhibits an inhibitory effect due to the catabolic repression in submerged fermentation (Manan and Webb 2017a). Solid-state fermentation can also be utilized for bio-detoxification of agricultural materials in order to eliminate the undesirable toxic compounds that may inhibit utilization of such nutrient-rich materials in a consequent microbial process. For example, Sharath et al. (2014) utilized solid-state fermentation as a bio-detoxification step to eliminate mainly phorbol esters in *Jatropha curcas* seed cake by fungal cultures.

There is a growing interest in solid-state fermentation at present from the biorefinery point of view. Researchers are trying to utilize solid-state fermentation for refining each and every fraction of the agricultural biomass as much as possible in order to approach the zero-waste mindset. For example, our group developed a strategy based on solid-state fermentation of wheat bran by *Aureobasidium pullulans* NRRL Y-2311-1 for the production of multiple value-added products, which have been sequentially extracted by a two-step extraction process (Yegin and Gelen 2025). In the first extraction, xylanase enzyme was recovered by using water as a solvent. Consequently, the enzyme-leached and dried solid residue, which had been accepted as a by-product of fermentation, was subjected to a second extraction step by methanol (80%, v/v) in order to recover phenolic compounds with high antioxidant capacity. This strategy provided simultaneous production and sequential recovery of multiple high-value products. In a study performed by Colla et al. (2023), solid-state fermentation of a soy bran and soy husk mixture was carried out by *A*. *niger* DAOM for the simultaneous production of proteases and peptides. Similarly, Leite et al. (2019) utilized different fungal strains comparatively in solid-state fermentation of various agricultural residues for the production of lignocellulolytic enzymes and antioxidant compounds. Unlike our strategy, in these studies, a typical single-step leaching process was performed, and the content of this extract was directly analyzed for different compounds. Summarizing, in addition to upgrading the nutritional status of cereals and legumes, solid-state fermentation is a very useful tool for the simultaneous production of multiple value-added products from plant-based materials within the biorefinery concept.

## Conclusion and future directions

Solid-state fermentation is one of the most ancient and widely utilized bioprocess modes for improving the nutritional status of food products, as evidenced by the increased content of bioactive phenolic and nitrogenous compounds, which have been documented in various studies. Solid-state bioprocess mode serves as a very effective and practical tool for the establishment of economically feasible and environmentally friendly routes to produce such types of compounds. Especially, achievements of high productivity and product stability with lower catabolic repression make this process much more attractive as compared to the submerged bioprocess mode. Moreover, it is more beneficial for the production and release of these bioactive compounds as compared to other techniques as well, such as enzyme-assisted extraction/modification, since it enables simultaneous biosynthesis of various microbial enzymes. Solid-state fermentation mode has also been widely used as a detoxification process to eliminate the undesirable toxic compounds from plant-based materials.

Despite the great potential of solid-state fermentation in the modification of plant-based materials, there are still several challenges that must be overcome in order to widen the application of this bioprocess mode at the industrial scale. The most critical issue is the design of the novel industrial bioreactor systems enabling more precise control of the fermentation parameters. More insights into microbial metabolism are required instead of randomly matching some common microbial strains with different plant-based solid substrates. By taking this into account, it might be possible to generate novel products with unique properties as a result of intentionally matching different combinations of microbial cell factories (e.g., co-culture systems and recombinant strains) and different mixtures of solid substrates under appropriate conditions. Utilization of microbial strains producing chimeric enzyme systems also possesses huge potential for identification of novel bioactive compounds. In the case of downstreaming, studies considering the development of efficient recovery and purification of such bioactive compounds are extremely scarce. Moreover, comprehensive in vivo and toxicological studies are required prior to the utilization of such products (e.g., phenolic extracts) with enhanced bioactive compounds for the benefit of human health. Further investigations to fill the mentioned gaps seem inevitable for future studies.

## Data Availability

Not applicable.
